# Influence of weevil on the physicochemical characteristics, functional properties, and nutritional value of rice (*Oryza sativa* L.) produced at Yagoua (far-north Cameroon)

**DOI:** 10.1016/j.heliyon.2024.e30918

**Published:** 2024-05-09

**Authors:** Serge Cyrille Houketchang Ndomou, Beatrice Tapita Balti, Stephano Tene Tambo, Marie Madeleine Nanga Ndjang, Katlafadaou Kaskawa, Christiant Kouebou, Hilaire Macaire Womeni

**Affiliations:** a CRESA Forêt-Bois, Faculty of Agronomy and Agricultural Science, University of Dschang, P.O Box 188, Yaounde, Cameroon; b Research Unit of Biochemistry, Medicinal Plants, Food Sciences and Nutrition, Department of Biochemistry, Faculty of Science, University of Dschang, P.O. Box 67, Dschang, Cameroon; c Society for the Expansion and Modernization of Rice Cultivation in Yagoua, P.O. Box 46, Yagoua, Cameroon; d Institute of Agricultural Research for Development, P.O. Box 415, Garoua, Cameroon

**Keywords:** Infested rice, Uninfested rice, Storage, Weevil, Functional properties, Nutritional quality

## Abstract

The infestation of rice by pests during the post-harvest phase is one of the consequences of the deterioration of its technological and nutritional quality. Therefore, the present study was carried out to determine the physico-chemical characteristics, functional properties and nutritional value of uninfested and weevil-infested rice during storage. To this end, rice samples were collected from a rice production unit in the Far North Region of Cameroon. The physico-chemical and functional properties of uninfested and infested rice flours were determined using standard methods. The results showed that among the functional properties, only porosity showed a significant difference (*p˂0.05*) between infested (13.88 %) and uninfested (17.30 %) rice flours. Concerning the proximate composition, except for the carbohydrate content, where a significant decrease (*p˂0.05*) was observed between infested (68.15 %) and non-infested (58.43 %) rice flours, no significant difference (p > 0.05) was observed for the other nutrients evaluated. It was also observed that weevil infestation had a significant (p < 0.05) effect on the mineral content of the rice samples. Furthermore, scanning electron microscopy analysis revealed the presence of smaller granules with imprecise shapes in non-infested rice flour. Thus, this study showed that weevil infestation had a significant negative impact on the nutritional quality of rice and that good practices must be adopted by producers to ensure the quality of rice grains during storage.

## 1 Introduction

Rice is the world's second most important cereal crop after maize, with nearly 510 million tonnes of milled rice produced worldwide in the last harvest year. By 2020, rice production in Asia will have increased to 4.8 t/ha, while Africa will have the lowest production yield, decreasing to 2.2 t/ha [1]. According to the Ministry of Agriculture, rice production in Cameroon in 2020 was estimated at 140,710 tonnes, while demand was estimated at 576,949 tonnes, resulting in 436,239 tonnes of imports to meet this excess demand [2]. In this context, the Government of Cameroon has adopted various strategies to develop rice production in order to improve productivity, ensure food security, meet the growing demand for agricultural raw materials by national industries, conquer international markets, particularly in African sub-regions, and reduce poverty [3].

However, one of the major and almost permanent constraints in the rice value chain is the storage system, which can affect the appearance and quality of the product. In fact, the quality of rice is a function of several parameters, namely climate, variety, cultivation techniques, weed control, post-harvest techniques (harvesting, storage, etc.) and the method of handling [4]. The infestation usually starts in the field, where the eggs are laid in the intact grains and the weevil develops its cycle completely inside the grains or grain fragments. The female lays up to 500 eggs inside the grain. The larvae feed on the protein and the insect only emerges as an adult. The development of this beetle takes between 25 and 35 days when the grain temperature is between 26 and 30 °C and the moisture content is 14 °C [5].

The rice weevil (*Sitophilus oryzae*) is one of the most dangerous pests of whole grains. Heavy infestations can cause spoilage and decomposition of stored products. The flour beetles doesn't just cause damage by feeding, but also cause further problems by contaminating the stored products, with large numbers of dead bodies, cast skins, and fecal pellets, as well as liquids secretions, and pungent odours in grain [6]. In addition, losses due to weevils alone can exceed 25 % of the crop and may even reach 40 % [7]. The development of these weevils also affects the physico-chemical properties of the resulting flours. Gabarty and Abou El Nour [8], showed a significant increase in the total protein contents and a decrease in monosaccharides and disaccharides in sieved and residual wheat flour samples infested with *Corcyra cephalonica, Ephestia kuehniella, and Tribolium confusum*. Therefore, this work aims to evaluate the physicochemical characteristics, functional properties, and nutritional value of rice infested by weelvil in a unit production at Yagoua, Cameroon.

## 2 Material and methods

### 2.1 Material

#### 2.1.1 Collection of samples

Once harvested, the paddy rice (IR46 variety) was sent for processing to a small-scale dehulling plant in the city of Yagoua. At the end of the transformation processes, the white rice obtained was then stored in warehouses for finished products to be collected. The number of bags to be sampled for a given batch was a function of the total number of bags [9]. The selection was made randomly, taking care to sample from the ends and the middle of the batch.

#### 2.1.2 Processing rice seeds into flour

Once collected, the white rice seeds were converted into flour according to the milling process described by Tambo et al. [10,11]. Indeed, the white rice was winnowed, sorted, ground and sieved (Ø = 300 μm) using a laboratory warring blender (POLYMIX KINETICA model, Japan). The rice flour obtained was packaged in polyethylene bags and then stored in a desiccator before use.

#### 2.1.3 Determination of the infestation rate

The infestation rate evaluates the proportion attacked in the store by the weevils and was measured using Equation (1) [9]:

(1) $$Infestation\;rate\;\left(\%\right)=\frac{Number\;of\;bags\;infested}{Total\;number\;of\;bags\;in\;the\;batch}*100$$

#### 2.1.4 Determination of functional properties of flour

##### 2.1.4.1 Determination of mass density, Hausner ratio, and porosity

The mass density, the Hausner ratio, and the porosity were determined according to the method described by Emeje et al. [12]. Indeed, twenty (20) grams of each sample powder was introduced into a 50 mL measuring cylinder and the volume occupied by the powders without disturbance was noted. Additionally, the sample with the measuring cylinder was tapped vigorously 100 times and the volume was noted. Bulk density, packing density, Hausner ratio, and porosity were calculated according to Equations (2), (3), (4):

(2) $$Bulk\;and\;tapped\;density=\frac{weight\;of\;sample\;\left(g\right)}{volume\;occupied\;by\;the\;sample}$$

(3) $$Hausner\;ratio=\frac{tapped\;density}{bulk\;density}$$

(4) $$Porosity\;\left(\%\right)=\frac{tapped\;density-bulk\;density}{tapped\;density\;}*100$$

##### 2.1.4.2 Determination of the swelling capacity

The methodology described by Okezie and Bello [13] was used to evaluate the swelling of flours. Indeed, 10 % (m/v) flour solutions were prepared and placed in a water bath (HWS-26, France) at 90 °C for 15 min. The mixture was then centrifuged at 4500 rpm for 15 min. The swelling rate was estimated as the difference between the mass of the sample having retained the water (M_1_) and that of departure (M_0_). The swelling rate was given by Equation (5):

(5) $$Swelling\;capacity\;\left(\%\right)=\frac{M1-M0}{M1}*100$$

##### 2.1.4.3 Determination of the water absorption capacity (WAC) and oil absorption capacity (OAC)

The WAC and OAC of different flour samples were evaluated according to the procedures described by Lin et al. [14]. Briefly, 10 % (W/V) flour solutions were prepared and placed in a water bath (HWS-26, France) at 90 °C. The mixture was centrifuged at 4500 rpm for 15 min. The volume of unabsorbed water or oil was measured. The WAC and OAC were given by Equation (6):

(6) $$WAC\;and\;OAC\;\left(\%\right)=\frac{Vi-Vf}{Vi}*100$$

where: Vi = Initial volume of water/oil, Vf = volume of water/oil after centrifugation.

#### 2.1.5 Evaluation of chemical characteristics of infested and uninfested rice flour

##### 2.1.5.1 Determination of anti-nutrient contents

The phytate content was determined by the method described by AOAC [15]. Two (2) g of sample were introduced into a 250 mL Erlenmeyer flask then 100 mL of 2 % HCL was introduced and the whole was left to stand for 3 h. The solution was then filtered using Whatman No. 3 paper. Fifty (50) mL of filtrate was introduced into a 250 mL Erlenmeyer flask with 107 mL of distilled water. Ten (10) mL of 0.3 % ammonium thiocyanate was added as an indicator. The whole was titrated with a standard solution of Fecl_3_, 6h_2_O which contains 0.00195 g of iron per mL. The phytate content was determined using the following Equation (7):

(7) $$Phytates\;content\;\left(\frac{mg}{100}g\right)=\frac{X*1.19}{0.195\;}*100$$

where: X the volume of the descent of the burette.

The oxalate content was determined by the method described by Day and Underwood [16]. A 1 g sample was taken and introduced into an Erlenmeyer flask, to which was added 75 mL of sulfuric acid H_2_SO_4_ of concentration 1.5 M; the mixture was stirred using a magnetic stirrer and then filtered using Whatman N° 1 filter paper. Twenty-five (25) mL of the filtrate were collected and introduced into a beaker then titrated hot (80–90 °C) with a solution of potassium permanganate KMnO_4_ at a concentration of 0.1 M until the solution turns to a persistent pink color for 30 s. The oxalate content was determined using the following Equation 8:

(8) %Oxalicacid=StandardValue*AverageTitre(0.02)

The determination of the saponin content was made by the method of Koziol [17]. A mass of 0.5 g of sample was introduced into a test tube, and 5 mL of distilled water was added to it. The tube was shaken vigorously for 30 s. Immediately after (5–10 s), the height of the foam formed was measured using a graduated ruler to the nearest 0.1 cm. The concentration of saponins was determined by the following Equation (9):

(9) $$Saponin\;content\;\left(\frac{mg}{100}g\right)=\frac{0.432*\left(foam\;height\;in\;cm+0.008\right)}{Mass\;ofl^{\prime }sample\;in\;g}*100$$

The method described by Gaytán-Martínez et al. [18] made it possible to quantify the tannins. For 1 g of powder of each sample, 25 mL of 1 % HCl (in methanol) was added. After 30 min of stirring, the mixture was centrifuged at 4000 rpm for 15 min and the supernatant was collected in another beaker. The residue was taken up and extracted twice in succession. The quantification of condensed tannins was done by mixing 1 mL of the collected extract with 5 mL of the reactive solution (50 g of vanillin and 8 mL of hydrochloric acid in 100 mL of distilled water) and the whole incubated at 30 °C for 20 min then the absorbance was read at 500 nm against a blank. On the other hand, the hydrolyzable tannins were read at 660 nm after mixing 500 μl of the extract with 1.75 mL of reactive solution (0.01 M FeCl_3_, 6h_2_O in 0.001 M HCl). The tannin content in the samples was calculated using a standard made from tannic acid solutions. The results were expressed in mg of tannic acid/100 g of dry matter.

##### 2.1.5.2 Determination of proximate composition

The proximate chemical composition of the different flours (moisture, protein, fiber, ash, lipid, carbohydrate contents, and energy value) was carried out according to the reference methodology described by AOAC [15].

##### 2.1.5.3 Determination of mineral content

The minerals contained in the different samples were quantified using atomic absorption spectrophotometry for calcium, magnesium, iron, and phosphorus and flame photometry for sodium and potassium [19].

#### 2.1.6 Scanning electron microscopy (SEM) analysis

The flours samples were defatted using the methods described by Indrani et al. [20]. To prevent the charge effect, the flours samples were mounted on aluminum stubs and spray-coated with a thin film of carbon using a carbon coater (Quorum Q150TE, Quorum Technologies, UK). After coating, the samples were transferred to the SEM specimen chamber and subjected to an electron beam under vacuum using a scanning electron microscope (Vega 3 XMU, TESCAN Czech Republic).

### 2.2 Statistical analyzes

The results were expressed by means ± standard deviations and were calculated using Microsoft Excel 2013 software. They were analyzed by the analysis of variance test (ANOVA) at the 5 % probability threshold and the two-tailed Fisher test was used to compare the means using the XLSTAT software, version 2021.

## 3 Results and discussion

### 3.1 Degree of infestation and impurities

Table 1 shows the level of infestation and contamination of rice samples during storage. Out of 167 randomly selected bags, a total of 6 bags of 50 kg were found to be infested with weevils, giving an infestation rate of 3.59 %. This finding could be caused by the temperature of the storage room, which was between 31 and 33 °C, and the humidity. These results were confirmed by Ndiaye [21], who showed that the conditions for the development of insects vary enormously, but most storage parasitic insects develop rapidly between 25 and 33 °C and 65 and 70 % relative humidity. The varieties used in the rice field are certainly known for their resistance to certain diseases, but the presence of weevils in the various stocks makes it clear that these varieties can be susceptible to insects. The weevil attacks the grain in the field and during storage. With regard to impurities, the value obtained in this study (0.60 %) is lower than the recommended value, which is 0.5 % [22].

**Table 1:** Infestation rate and impurity rate.

Parameters	Values (%)	Recommended values (%)
Infestation rate	3.59	/
Impurity rate	0.60	0.50

### 3.2 Functional properties of infested and non-infested rice flour

The physical and functional properties define the usefulness, application, packaging and transport conditions of a food. The results of the bulk density, porosity, and Hausner ratio as well as the swelling rate, and water and oil absorption capacity of different rice samples are presented in Table 2. It appears that except for the porosity which showed a significant difference (*p˂0.05*) between infested rice (13.88 ± 0.41 %) and uninfested rice (17.30 ± 1.84 %), all other parameters showed no significant difference between samples. The difference observed in porosity could be due to the elimination of air bubbles by the presence of weevils. It could also be linked to the lower protein content in uninfested flour, as shown by the work of Tambo et al. [10]. In fact, porosity is one of the key parameters in determining the storage, transport, and packaging conditions of food products [23]. It measures the voids between solid particles of a substance, and the pores can be filled with gas or water.

**Table 2:** Physical and Functional properties of infested rice and uninfested rice flour.

parameters	IR	UR
**Swelling capacity (%)**	56.59 ± 1.46^a^	51.61 ± 2.96^a^
**WAC (%)**	3.50 ± 0.71^a^	4.40 ± 0.57^a^
**OAC (%)**	15.00 ± 1.41^b^	25.00 ± 1.41^a^
**Mass density (g/ml)**	0.79 ± 0.01^a^	0.77 ± 0.02^a^
**Taped mass density (g/ml)**	0.92 ± 0.01^a^	0.95 ± 0.01^a^
**Porosity (%)**	13.88 ± 0.41^b^	17.30 ± 1.841^a^
**Ratio Hausner**	1.16 ± 0.01^a^	1.20 ± 0.03^a^

**RI**: infested rice; **UR**: uninfested rice. The results are the means ± the standard deviation of 2 repetitions. Values in the same line and followed by the same letter are not significantly different (p > 0.05). WAC: water absorption capacity; OAC: oil absorption capacity.

The values of the Hausner ratio obtained in this study were 1.16 and 1.20 for infested and uninfested rice respectively. These values are not far from those found by Santomaso et al. [24] who reported that a Hausner ratio of 1–1.25 identifies powders with excellent and almost free flow. However, according to Fitzpatrick [25], a Hausner ratio greater than 1.25 generally indicates fairly fluid powder behaviour.

Bulk density analysis also showed a similarity between the infested (0.79 g/mL) and uninfested rice (0.77 g/mL) respectively (p > 0.05). This parameter is related to the protein composition and granule size of the samples [26]. It does not the same as the observations made by Tambo et al. [10,11] who showed a positive correlation between this parameter and protein content. These results are greater than those obtained by Tambo et al. [11] in maize and cassava flours. The high protein content of rice could explain these differences. These results also show that these flours are more suitable for cake formulation than as supplementary feeds [26].

The water absorption capacity (WAC) and the swelling rate reflect the hydration capacity of the dough, in the presence of liquid water, and depend mainly on the humidity, and the rate of starch damage [27]. It is related to the molecular structure and the chemical composition of the starch [28]. The results obtained concerning the WAC for infested rice and uninfested rice were 3.50 % and 4.40 %, respectively. On the other hand, they were 56.59 % and 51.61 % for infested and uninfested rice, respectively, for the swelling capacity. This discrepancy could be due to the difference in amylose and amylopectin content of the two flours since the weevils feed on the nutrients of the rice such as carbohydrates, the first source of energy. In addition, it could be related to a decomplexation of starch molecules during degradation by weevils, thus facilitating water retention. Similarly, the presence of soluble amino acids in insect proteins would also be a reason for the greater water retention and swelling potential observed in infested rice.

Table 2 also shows that the infestation significantly (*p˂0.05*) reduced the oil absorption capacity (OAC). Indeed, the germ is the main reserve of nutrients in the seed. These chemical elements, as well as proteins and lipids, can interact with hydrophobic molecules to give umami properties to these flours. This is further explained by the higher water retention capacity of infested seeds. In fact, these two parameters are negatively correlated [29]. In addition, the presence of polar amino acids in the insect carcass would also be responsible for this observed difference. The OACs in the two flours are in the range of those obtained by Klang et al. [29] on potato flours.

### 3.3 Antinutrient content

Table 3 shows the results of the antinutrient content of infested and uninfested rice. In both samples, the analysis of the antinutrients (condensed tannins, hydrolyzable tannins, oxalates, phytates, saponins) showed no significant differences (*p˃0.05*). The values obtained are also below the maximum standard for phytates (less than 250 mg/100 g of food) and oxalates (less than 450 mg/100 g of food), thus making rice a source of divalent cations. In view of the results obtained, this low rate could be explained by the fact that adult weevils attack the plants in the rice field by as they approach maturity, preventing the synthesis of these secondary metabolites as a defence mechanism harvesting. According to Krief [30], plant secondary metabolites are known for their defensive role against predators and pathogens. However, these defence molecules exist in an inactive form and only become toxic when attacked [31]. The presence of antinutritional substances (tannins, phytates, oxalates, hydrocyanic acid, and thiaminase) in the digestive juices of certain insects such as bees, may also explain this variation [32]. However, the values obtained for phytate (83.23 mg/100 g) are nonetheless lower than those of Fotso et al. [33] on optimized soy flour (*Glycine* max L.). Phytate reduces the bioavailability of minerals, and the solubility, functionality, and digestibility of proteins and carbohydrates [34]. Tannin-protein complexes can cause the inactivation of digestive enzymes and reduce protein digestibility through the interaction of the protein substrate with ionizable iron [35]. The presence of tannins in food can therefore reduce feed efficiency, depress growth, reduce iron absorption, damage gastrointestinal mucosa, alter the cation excretion and increase protein and essential amino acid excretion [36]. Dehulling, cooking, and fermentation reduce the tannin content of cereals and other foods.

**Table 3:** Antinutrient content of infested and uninfested rice flour.

parameters	IR	UR
**Condensed tannins (mg/100g)**	3.30 ± 0.03^a^	1.88 ± 0.08^a^
**Condensed hydrolyzable tannins (mg/100g)**	0.48 ± 0.00^a^	0.17 ± 0.00^b^
**Oxalate (mg/100g)**	4.50 ± 1.53^a^	3.75 ± 1.29^a^
**Phytates (mg/100g)**	18.30 ± 0.00^a^	14.23 ± 3.52^a^
**Saponins (mg/100g)**	0.40 ± 0.06^a^	0.49 ± 0.06^a^

**IR**: infested rice; **UR**: uninfested rice. The results are the means ± the standard deviation of 2 repetitions. Values in the same line and followed by the same letter are not significantly different (p > 0.05).

### 3.4 Proximate composition of infested and uninfested rice flour

Table 4 highlights the proximate composition of the infested and uninfested rice. For the moisture content, the values obtained (8.50 % and 7.50 % for infested and non-infested rice, respectively) are lower than the 15 % recommended by the Codex Alimentarius for rice [22]. According to Ndangui [37], the moisture content is very important in assessing the preservation and stability of a product during storage. However, the high values observed in infested rice would be related to the destruction of the pectocellulosic wall by weevils, thus facilitating water uptake. This water uptake is also due to a combination of water molecules and released carbohydrates via hydrogen bonds. In fact, Kumarakuru et al. [38] have demonstrated that additional water uptake of flours is observed during food processing, as a result of the degradation of macromolecules that are more apt to bind these molecules present in the ambient air. In addition to this, the presence of insects in a bag of rice would indicate poor storage and the presence of holes, which would also lead to an increase in the hygroscopicity of the rice grains. The values obtained are higher than those found by Tambo et al. [39] in sprouted maize meal. These results demonstrate to the poor storage of rice sacks, the use of leaky sacks as packaging and the losses that could result from infestation by these insects. These losses could lead to shortage that could cause famine.

**Table 4:** Proximate composition of infested and uninfested rice flour.

Parameters	IR	UR
**Moisture content (%)**	8.50 ± 0.70^a^	7.50 ± 0.70^a^
**Protein content (%)**	19.25 ± 0.01^a^	13.13 ± 0.01^b^
**Lipid content (%)**	2.08 ± 0.13^a^	2.46 ± 0.42^a^
**Ash content (%)**	1.00 ± 0.00^a^	1.00 ± 0.00^a^
**Fiber content (%)**	10.72 ± 0.14^a^	7.75 ± 0.14^b^
**Carbohydrate content (%)**	58.43 ± 0.69^b^	68.15 ± 0.98^a^
**Energy value (Kcal)**	329.56 ± 1.57^b^	347.33 ± 0.13^a^

**IR**: infested rice; **UR**: uninfested rice. The results are the means ± the standard deviation of 2 repetitions. Values in the same row and followed by the same letter are not significantly different (p > 0.05).

Proteins play many important biological and physiological roles, such as enzyme, energy sources, membrane and tissue components, and hormones. Regarding the protein content, the values obtained for infested rice (19.25 %) and for uninfested rice (13.13 %) are in agreement with those of Samuels and Modgil [40] who demonstrated that an increase in insect infestation and storage time significantly increased the protein content of wheat during storage. Moreover, Ahmedani et al. [41] reported an increase in the total protein content of wheat varieties six months after infestation by *Trogoderma granarium.* In fact, the insects were not removed from infested rice during milling prior to analysis, which could explain this high rate since insects are a source of protein in the order of 45–75 %. The values obtained by Kumarakuru et al. [38] are higher than those in this study (between 16.70 and 21.10 %). This can be explained by the fact that cereals are not primarily source of protein. Nevertheless, the values obtained in this study would contribute to more than 100 % of the recommended daily protein requirement for two-year-old children, even though the consumption of infested rice is not recommended [42].

Regarding the lipid content, this study recorded values of 2.08 % and 2.46 % for infested rice and uninfested rice respectively (Table 4). These results are in contrast to those obtained by Juliano and Goddard [43], who showed that milled rice contains 1.50–1.70 % lipid mainly in the form of non-starch lipids. However, Rahanitrarivony [44] revealed that the lipid content of brown rice varies from 0.60 to 4.00 %, while milled rice contains only 0.20–2.70 %, and that, depending on the milling method pounded rice has a higher fat content than milled rice. This high rate could therefore be explained by the degree of processing. Moreover, certain organoleptic properties of which the IR 46 variety, such as the shiny texture after cooking, could be related to lipids. This is confirmed by the work of Ferron and Guichard [45], who studied the role and influence of lipids on the formation, release, and perception of aroma compounds. They estimated that fat can be at the origin of the formation of volatile compounds, mainly resulting from the phenomena of oxidation of polyunsaturated fatty acids. Although the variation is not significant, the low lipid content obtained in the infested rice indicates a loss of this nutrient as a result of the destruction by the insects of the germ, where the nutrients are stored. The values obtained also suggest that rice should be supplemented with legumes before use, to reduce the risk of malnutrition.

Carbohydrates are the main source of energy directly metabolizable by the brain, providing 4 kcal/g [46]. For the total carbohydrate content, there was a significant difference (*p˂0.05*) between infested rice (58.43 %) and uninfested rice (68.15 %). Several authors have observed a significant decrease in the carbohydrate content of grains following insect infestation. In fact, Jood et al. [47] found a significant reduction in the carbohydrate content of wheat, maize, and sorghum artificially infested with *T. granarium* and *R. dominica*. Furthermore, Singh et al. [48] reported that insect infestation by *Rhizopertha dominica* induces a 13.50 % reduction in the total carbohydrate content. These results were also confirmed by Hameed et al. [49], who observed a significant reduction in the carbohydrate content of wheat grains due to the attack of the larvae of *T. granarium.* The high carbohydrate content in normal rice would also be related to its lower protein content. Indeed, Tambo et al. [10,11] reported a negative correlation between these two parameters. The contents obtained are lower than the 73–82 % range obtained by Eke-Ejiofor and Friday [50] on wheat-based flours. This demonstrates a negative effect of infestation on this important parameter for the techno-functional properties of cereal-based foods. According to the Canadian Grain Commission [5], storage pests feed on grains or flours mainly made up of carbohydrates which play the role of primary energy source for living beings. These insects mainly attack the starch-rich seed germ, leading to a reduction in starch and hence carbohydrate content. In addition, partial hydrolysis of the osidic bonds of starch by the amylases secreted by the insect pests would also lead to an increase in simple sugar content. These results also show that this cereal (infested or not) cannot be used in the formulation of snacks, as its carbohydrate content is less than 70 %.

Fibres are responsible for a number of beneficial physiological effects, such as improving intestinal transit speed to reduce the occurrence of cancer [51]. Evaluation of the fiber content showed that they were significantly *(p˂0.05)* higher in the infested rice (10.75 %). This can be explained by the degradation of the germ, especially the starch stored there, since the pectocellulose wall is rich in cellulose and cellobiose which are difficult to digest. These results contradict those of Ahmedani et al. [41] who reported a decrease in the fiber content of weevil rice as a result of the destruction of walls, which are essentially rich in fiber. However, the fiber contents obtained in this study are higher than those found in paddy rice by Klang et al. [52], which could be explained by the difference in the variety of rice used.

The ash content is related to the mineral intake and thus determines the potential of the feed for future use in the fight against hidden hunger [53]. The evaluation of the ash content showed that it is less important than the 3 % found in Paddy rice by Klang et al. [52]. A varietal difference related to mineral loss after weevil infestation would explain this difference. However, the values obtained are remains within the recommended range (1–3 %).

The energy value is related to the nutrient composition especially glucid, lipid and protein content. Tambo et al. [10] reported a significant positive relationship between energy value and carbohydrate, fat and protein content. The values obtained show that the uninfested rice is more energetic because it is richer in total nutrients (glucids). These results demonstrate the importance of proper food preservation to limit spoilage and loss of energy-giving nutrients.

### 3.5 Mineral composition of infested and uninfested rice flour

Table 5 indicates the contents of some minerals detected in infested rice and uninfested rice. There was a significant difference (*p˂0.05*) between potassium; sodium; magnesium and calcium contents of the two samples, with the highest values recorded in the infested rice. This could be associated to the high mineral content of the weevils, as they were not eliminated during the production of the infested rice powders. The table also shows that calcium and potassium levels were highest in both samples. This is in agreement with the work of Olaleye et al. [54] and Chelangat et al. [55], who found high levels of these minerals in bambara groundnut. Minerals are essential in human health as they play important roles in physiological functions, enzyme synthesis, hormones, growth regulation, boosting the immune and reproductive systems [56]. However, a deficiency in the micronutrients in human diet can lead to health problems such as anaemia, nervous system damage, and heart disease [57]. Table 5 also revealed that potassium (K) concentration to be higher than sodium (Na), the Na/K ratio in the diet is less than 1, which is an important factor in the prevention of hypertension. The mineral contents obtained in this study are much lower than those reported by Diallo et al. [58] for Ca, Na, K, Mg and P. The present study showed a Na/K ratio of 0.25, which is within the range reported by Rainakari et al. [59]. However, in this study, the calcium concentration was higher than the phosphorus concentration, with a Ca/P ratio greater than 1. According to Belitz et al. [60], the Ca/P ratio in the diet is be equal to or less than 1 to ensure their good absorption by the organism. In general, the levels of minerals assessed in this study are lower than their Recommended Daily Allowances (RDA). The use of these flours to fight hidden hunger should therefore be combined with other foods such as moringa before being used in the preparation of supplementary feeds. These results also suggest that infestation is beneficial overall in terms of mineral content, although the toxicological effects remain questionable.

**Table 5:** Mineral composition of infested and uninfested rice flour.

Samples	IR	UR	RDA (min)	RDA (max)
**Fe (mg/100g)**	2.04 ± 0.01^a^	2.04 ± 0.01^a^	2.1	28
**P (mg/100g)**	44.36 ± 0.01^a^	31.00 ± 0.07^b^	120	1600
**Mg (mg/100g)**	87.53 ± 0.01^a^	58.37 ± 0.01^b^	45	450
**K (mg/100g)**	206.79 ± 0.10^a^	80.860 ± 0.10^b^	600	6000
**Na (mg/100g)**	52.78 ± 0.10^a^	20.50 ± 0.10^b^	375	5000
**Ca (mg/100g)**	352.50 ± 0.10^b^	400.50 ± 0.10^a^	120	1600
**Na/K**	0.25	0.25		
**Ca/P**	7.94	12.91		

**RDA:** Recommanded Daily Allowances; **Max:** Maximum; **Min:** Minimum; **IR**: infested rice; **UR**: uninfested rice. The results are the means ± the standard deviation of 2 repetitions. Values in the same row and followed by the same letter are not significantly different (p > 0.05).

### 3.6 Starch granule structure of infested and uninfested rice

**Fig. 1:**
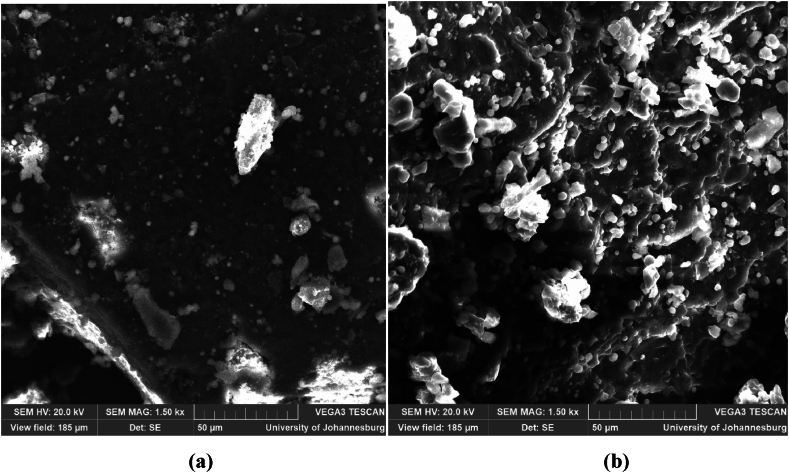
Effect of weevil attack on the structure of rice flour. a: infested rice; b: uninfested rice.

Fig. 1 shows the starch granule structure of infested and uninfested rice. It can be observed that the infested rice (Fig. 1a) had granules of smaller size and imprecise shape unlike those of the uninfested rice, which were mostly spherical and ovoid shape (Fig. 1b). This difference could be explained by the destruction of the germ source of the starch reserve by the insects. The more compact structure of uninfested rice would also be linked to the formation of complexes between lipids, proteins, and carbohydrates [61]. We also note the presence of dark areas at many points. This could be explained by the loss of water during drying [62]. These areas are more pronounced in infested flours, thus showing a rapid loss of moisture in proportion to its absorption. The gelatinous structure observed in Fig. 1b also testifies to the good water retention capacity which is directly related to the content of hydrophilic amino acids and starch [63]. The shiny appearance of the gels is linked to the higher lipid content in uninfested rice and its greater capacity to retain oil.

## 4 Conclusion

It appears from this study that the infestation of rice grains is influenced by post-harvest, drying, and processing conditions. When functional properties of rice samples were analyzed, only the porosity was affected by the weevil activity. Furthermore, the proximate chemical characterization revealed that the carbohydrate content and the energy value of the infested rice dropped considerably. Furthermore, the anti-nutrient levels assessed in this study showed that the weevil activity increased these parameters in rice. Careful control of production and storage conditions would limit the harmful effects on rice samples and provide added value, particularly in the formulation of many products.

## 5 Novelty of the work

• The influence of infestation on the physico-chemical and functional properties and structure of rice seeds has been demonstrated.
• Weevils infestation of rice reduces carbohydrate content, increases water content, reduces shelf life and leads to loss of starch structure.
• Pest infestation increases anti-nutrient content and reduces calcium availability.

## 6 Perspective

This article gives an initial idea of the harmful effects of larvae and insect attack on the physico-chemical, functional and anti-nutritional properties of rice when stored incorrectly. Above all, it will show people the nutritional losses they will suffer if they consume such a foodstuff. To make this work more attractive, we will take this study a step further by assessing the degradation kinetics of rice's main constituent, starch, and the prolonged effect of exposure time to rice pests on the formation of toxic agents such as afflatoxins, heavy metals and other chemical compounds. Finally, studies will be carried out on the toxicity of consuming such a food *in vitro* and *in vivo*.

## Originality of work

The authors declare that this work is original and has not been submitted anywhere.

## Additional information

No additional information is available for this paper..

## Funding

This study didn't receive any funding.

## Ethical approval

This article does not contain any studies with human or animal participants performed by any of the authors.

## Data availability statement

All the data that support the conclusions will be made available on request.

## CRediT authorship contribution statement

**Serge Cyrille Houketchang Ndomou:** Writing – review & editing, Writing – original draft, Visualization, Validation, Software, Project administration, Methodology, Investigation, Formal analysis, Data curation, Conceptualization. **Beatrice Tapita Balti:** Writing – review & editing, Writing – original draft, Visualization, Validation, Resources, Methodology, Investigation, Formal analysis, Conceptualization. **Stephano Tene Tambo:** Writing – review & editing, Writing – original draft, Validation, Methodology, Investigation, Data curation. **Marie Madeleine Nanga Ndjang:** Writing – review & editing, Writing – original draft, Methodology. **Katlafadaou Kaskawa:** Writing – review & editing, Methodology, Formal analysis. **Christiant Kouebou:** Writing – review & editing, Methodology, Formal analysis. **Hilaire Macaire Womeni:** Writing – review & editing, Visualization, Supervision, Project administration.

## Declaration of competing interest

The authors declare the following financial interests/personal relationships which may be considered as potential competing interests:Stephano Tambo TENE and Serge Cyrille Houketchang Ndomou report administrative support, equipment, drugs, or supplies, and writing assistance were provided by University of Dschang Department of Biochemistry. If there are other authors, they declare that they have no known competing financial interests or personal relationships that could have appeared to influence the work reported in this paper.
